# Effmann Type IIA2 Urethral Duplication with Anorectal Malformation Complicated by Posterior Urethral Diverticulum: A Staged Minimally Invasive Approach

**DOI:** 10.70352/scrj.cr.26-0496

**Published:** 2026-09-04

**Authors:** Shunya Takada, Hiroo Uchida, Chiyoe Shirota, Takahisa Tainaka, Satoshi Makita, Akihiro Yasui, Aitaro Takimoto, Kaito Hayashi, Daiki Kato, Hiroki Ishii, Hajime Asai, Kazuki Ota

**Affiliations:** Department of Pediatric Surgery, Nagoya University Graduate School of Medicine, Nagoya, Aichi, Japan

**Keywords:** urethral duplication, anorectal malformation, neonatal urinary retention, rectourethral fistula, prostatic utricle, laparoscopic-assisted anorectoplasty, posterior urethral diverticulum

## Abstract

**INTRODUCTION:**

Urethral duplication associated with anorectal malformation (ARM) is rare and can make both diagnosis and surgical reconstruction difficult.

**CASE PRESENTATION:**

A male infant was born at 31 weeks’ gestation, weighing 1362 g, with multiple congenital anomalies, including duodenal atresia, right renal agenesis, a tethered cord with a terminal filar lipoma, and a ventricular septal defect. He developed neonatal urinary retention; urethral drainage was possible only with a fine intravenous catheter, and when this became obstructed, a percutaneous cystostomy was placed. Contrast studies demonstrated intermediate-type ARM with a rectobulbar urethral fistula, an enlarged prostatic utricle, and complete sagittal urethral duplication (Effmann type IIA2). Distal to the bulbar urethra, the urethra divided into a severely stenotic dorsal channel and a ventral channel that carried voiding but was also stenotic. At 8 months of age, laparoscopic-assisted anorectoplasty (LAARP) was performed with excision of the prostatic utricle; the rectourethral fistula was divided on the rectal side to avoid urethral injury, and the retained remnant formed an acquired posterior urethral diverticulum (PUD). The residual stenosis and the PUD were then managed endoscopically by balloon dilation of the ventral channel and holmium:yttrium-aluminum-garnet laser ablation of the PUD. The dilation restored spontaneous voiding and allowed cystostomy removal at 21 months of age without open urethral reconstruction. At the most recent follow-up (age 28 months), the patient maintains spontaneous voiding with preserved renal function.

**CONCLUSIONS:**

We encountered ARM associated with urethral duplication in which both urethral channels were stenotic and caused neonatal urinary retention. Early urinary diversion allowed accurate anatomic assessment, and the functional ventral channel was preserved by keeping the fistula dissection on the rectal side during LAARP. Staged endoscopic management of the residual stenosis and the acquired PUD achieved stable spontaneous voiding without extensive open urethral reconstruction.

## Abbreviations

ARM
: anorectal malformation
LAARP
: laparoscopic-assisted anorectoplasty
PUD
: posterior urethral diverticulum
VCUG
: voiding cystourethrography
YAG
: yttrium-aluminum-garnet

## INTRODUCTION

Urethral duplication is a rare congenital anomaly with variable anatomy and clinical presentations that range from incidental findings to urinary obstruction, recurrent infection, or a double urinary stream.^1)^ The classification proposed by Effmann et al., which is based on cystourethrographic anatomy and distinguishes incomplete (type I) from complete (type II) duplications, is widely used to describe these anomalies.^2)^ In a systematic review of urethral duplication in boys, complete sagittal duplications—Effmann type IIA2 and its Y-variant (IIA2-Y)—accounted for 58.6% of reported cases^1)^; in type IIA2 the 2 channels share a common bladder origin and lie in the mid-sagittal plane.^2)^ The ventral channel is usually the functional one, whereas the dorsal (orthotopic) channel is usually narrow or poorly developed,^2)^ and the degree of dorsal patency largely determines the reconstructive options.^3)^ Narrowing of the ventral channel itself is uncommon; however, when both channels are narrowed, the bladder cannot empty, placing renal function at risk.^1)^

ARM is often accompanied by urological anomalies, warranting systematic evaluation of the urinary tract as a part of the initial workup.^4)^ However, the coexistence of ARM and urethral duplication is exceptionally rare and is largely confined to the complete duplication types, documented primarily in isolated case reports or small case series.^1,5,6)^ This combination raises the risk of diagnostic oversight and of iatrogenic urethral injury during definitive anorectal reconstruction, particularly when the duplication is not recognized preoperatively.^7)^

Herein, we report an infant with complete sagittal urethral duplication (Effmann type IIA2, without a Y-configuration), an intermediate-type ARM with a rectourethral fistula, and narrowing of both urethral channels. Neither channel allowed urine to pass, and he presented as a neonate with complete urinary retention that could not be relieved by conventional urethral catheterization; this presentation is rarely reported.^1)^ We describe the preoperative diagnostic work-up and a staged, minimally invasive management strategy for this rare combination, including endoscopic treatment of an acquired PUD that resulted from protecting the functional urethra during anorectoplasty.

## CASE PRESENTATION

Antenatally, serial fetal ultrasonography showed no bladder distension, hydronephrosis, hydroureter, or urethral dilatation, and there was no oligohydramnios; a mild tendency toward polyhydramnios was noted at 27 weeks’ gestation, and fetal MRI was not performed. A male infant was delivered at 31 weeks of gestation with a birth weight of 1362 g. Initial postnatal evaluation revealed multiple congenital anomalies, including duodenal atresia, right renal agenesis, spinal anomalies (tethered cord with terminal filar lipoma), and a ventricular septal defect. The external genitalia were of male type, with an imperforate anus and no perineal fistula; no urethral meatus was visible at the tip of the penis, and both testes were undescended. He voided spontaneously about 2 hours after birth. Renal and bladder ultrasonography on postnatal day 1 showed no bladder-wall thickening, no hydronephrosis, no ureteric dilatation, and no dilatation of the lower urinary tract; the right kidney could not be identified and has not been demonstrated on any subsequent study.

On postnatal day 3, continuous intravenous fentanyl (1.5 µg/kg/h) was started for sedation because bleeding from the decompression tube placed for the duodenal atresia raised the suspicion of gastrointestinal ulceration. On postnatal day 4, he developed progressive abdominal distension and acute urinary retention, with a distended bladder on abdominal ultrasonography. Initial attempts at urethral catheterization were unsuccessful, even with a 3-Fr catheter, due to severe distal urethral narrowing; urine was finally drained through a 27-gauge peripheral intravenous catheter passed per urethra, and fentanyl was then tapered. On postnatal day 6, the catheter was found to be obstructed, with urine again accumulating in the bladder; fentanyl was discontinued completely, and the catheter was removed. No spontaneous voiding followed, and repeated attempts at catheterization, with the same 27-gauge catheter and with a guidewire, failed. Retrograde urethrography showed that contrast stopped at the bulbar urethra and that the bladder did not opacify. A suprapubic cystostomy was therefore placed on postnatal day 7 during the same operative session as the duodenoduodenostomy and transverse colostomy. The diversion was a percutaneous, US-guided cystostomy rather than a surgical vesicostomy; an indwelling 6-Fr balloon catheter was placed and changed monthly until its removal at 21 months of age. A catheter-free vesicostomy was deliberately avoided because the infant was extremely preterm and of very low birth weight with multiple anomalies requiring staged operations and because the stenotic duplicated urethra was expected to be amenable to endoscopic recanalization, allowing eventual catheter removal without committing the child to a second diversion and its later closure.

**Fig. 1:**
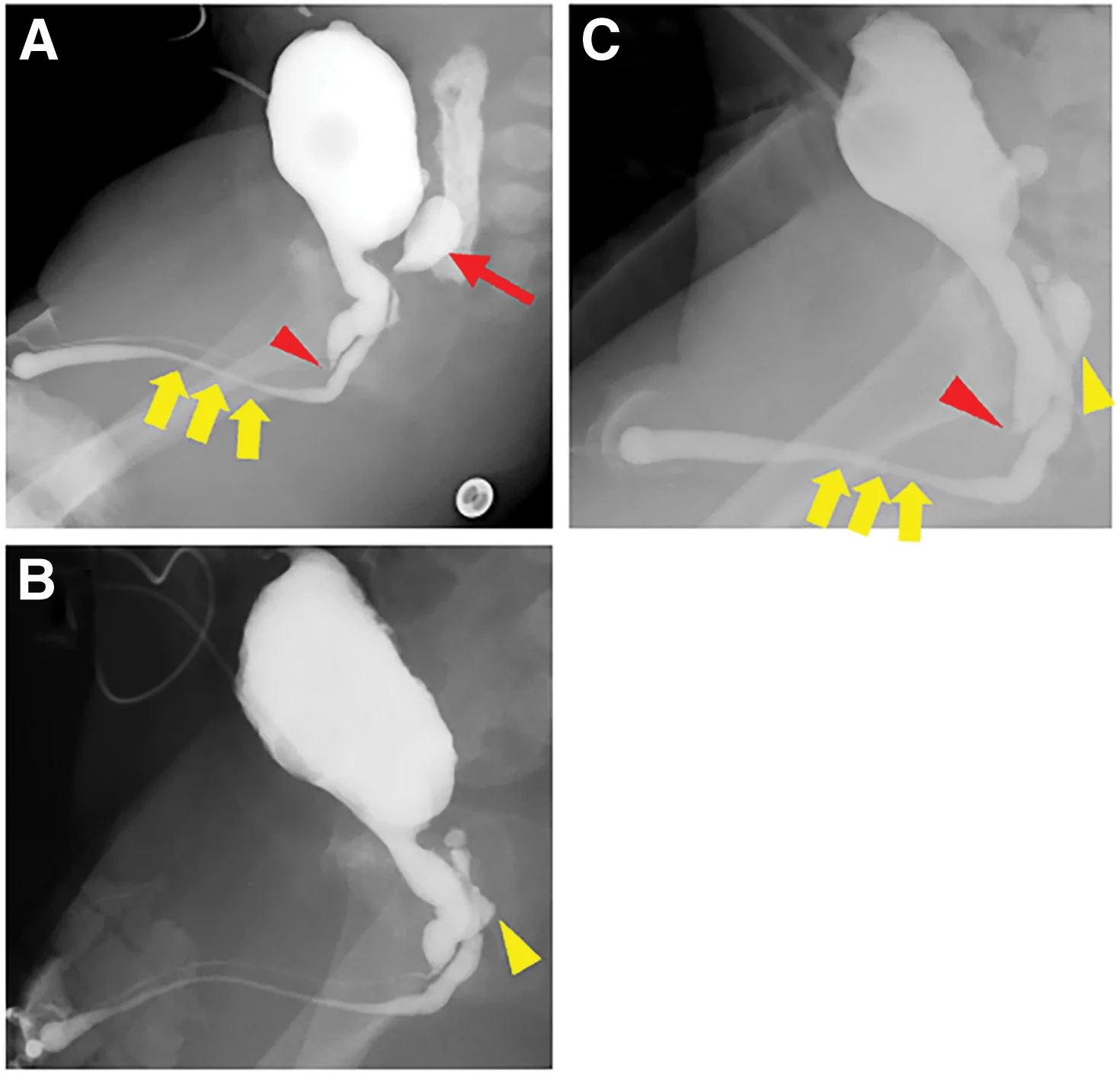
VCUG findings. (**A**) Preoperative: enlarged prostatic utricle (red arrow); bifurcation into a stenotic dorsal channel (red arrowhead) and a ventral voiding channel with distal stenosis (yellow arrows). (**B**) Postoperative, before transurethral treatment: acquired PUD at the site of the divided rectourethral fistula (yellow arrowhead). (**C**) At cystostomy removal (21 months of age): voiding through the ventral channel, with improvement of the stenosis (yellow arrows); the dorsal channel is occluded (red arrowhead), and a small residual PUD is present (yellow arrowhead). PUD, posterior urethral diverticulum; VCUG, voiding cystourethrography

On genital examination during the neonatal period, there was a preputial adhesion, and no urethral meatus could be seen at the tip of the penis; the ectopic ventral meatus was not apparent at this stage, so that the duplication was recognized on contrast studies rather than on inspection. Subsequent imaging—including VCUG via the cystostomy and distal colostography—demonstrated intermediate-type ARM with a rectobulbar urethral fistula and an enlarged prostatic utricle. Crucially, VCUG confirmed complete sagittal urethral duplication (Effmann type IIA2) (**Fig. 1A**). The utricle measured 8.7 × 18.7 mm, whereas the rectourethral fistula entered the bulbar urethra. The urethra bifurcated distal to the bulbar urethra into 2 channels that lay in the same mid-sagittal plane, with the orthotopic urethra lying dorsally and the accessory urethra ventrally. On VCUG, the dorsal channel was stenotic, whereas voiding occurred through the ventral channel; stenosis was also observed from the ventral channel to the bulbar urethra. There was no vesicoureteral reflux. Before LAARP, the cystostomy catheter was kept unclamped, and a small amount of spontaneous voiding was nonetheless observed.

**Fig. 2:**
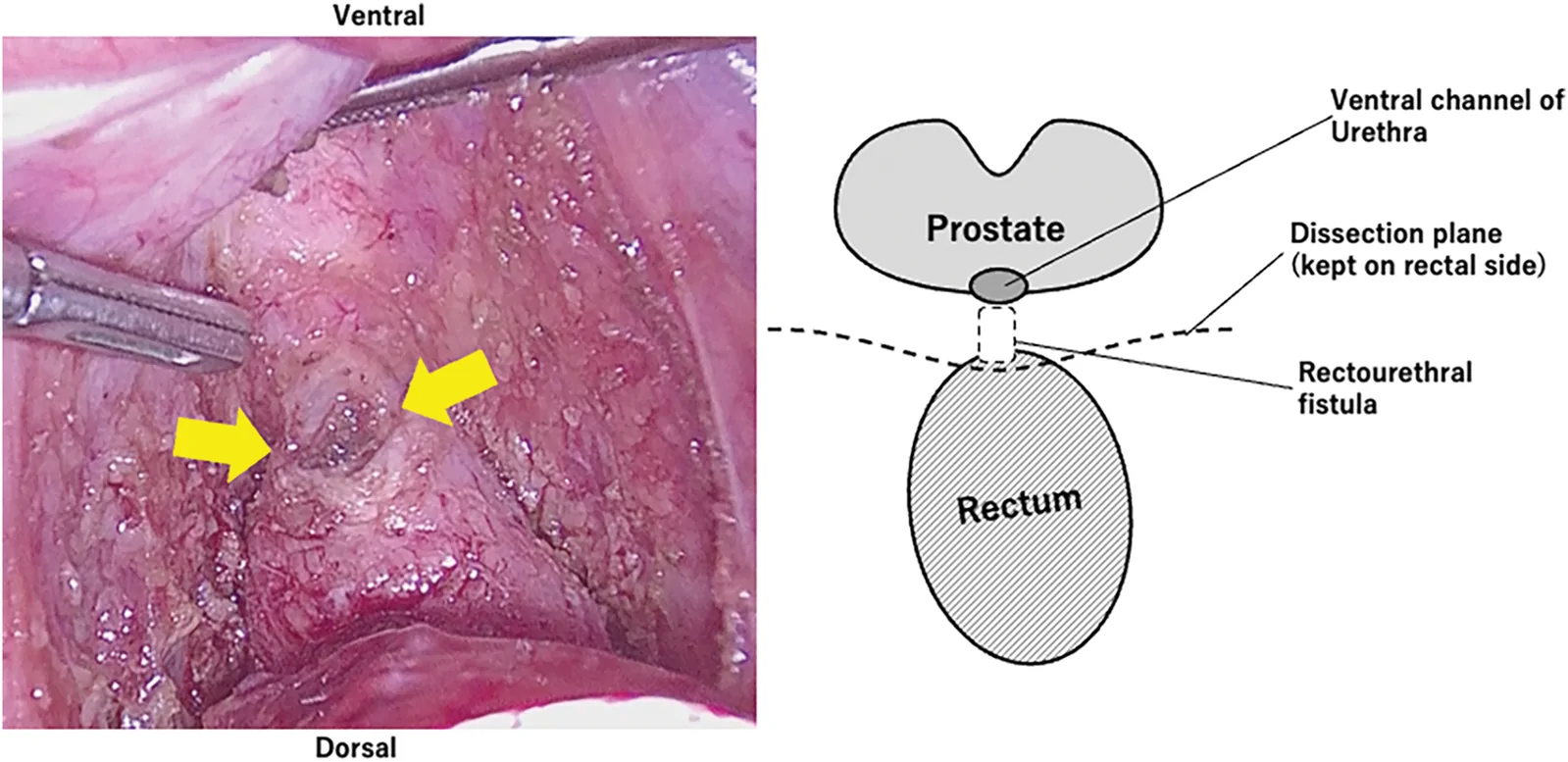
Intraoperative view during LAARP and schematic illustration. Division and ligation of the rectourethral communication: the dissection plane was kept on the rectal side to protect the functional ventral urethral channel. The yellow arrows indicate the site of fistula division on the rectal side. LAARP, laparoscopic-assisted anorectoplasty

At 8 months of age, after sufficient weight gain, he underwent LAARP for ARM repair. Intraoperative urethroscopy showed a mid-urethral stenosis, and the rectourethral fistula itself could not be directly visualized. Because the ventral channel was the functional voiding pathway, it was protected by dividing the rectum and ligating its fistulous communication without approaching the urethra, keeping the dissection plane on the rectal side (**Fig. 2**). The enlarged prostatic utricle was ligated at its prostatic attachment and completely excised. No urethral injury occurred intraoperatively or postoperatively.

On the postoperative VCUG obtained before any transurethral treatment (**Fig. 1B**), the prostatic utricle had been completely resected, but a new outpouching was seen at the bulbar urethra at the site of the divided rectourethral fistula; this remnant of the fistula constituted an acquired PUD, which measured 13.7 × 2.7 mm. On voiding, the stenosis of the ventral channel and of the bulbar urethra was unchanged from its preoperative appearance, indicating that the functional ventral channel had been preserved. Spontaneous urethral voiding accordingly remained inefficient, with significant post-void residual urine.

**Fig. 3:**
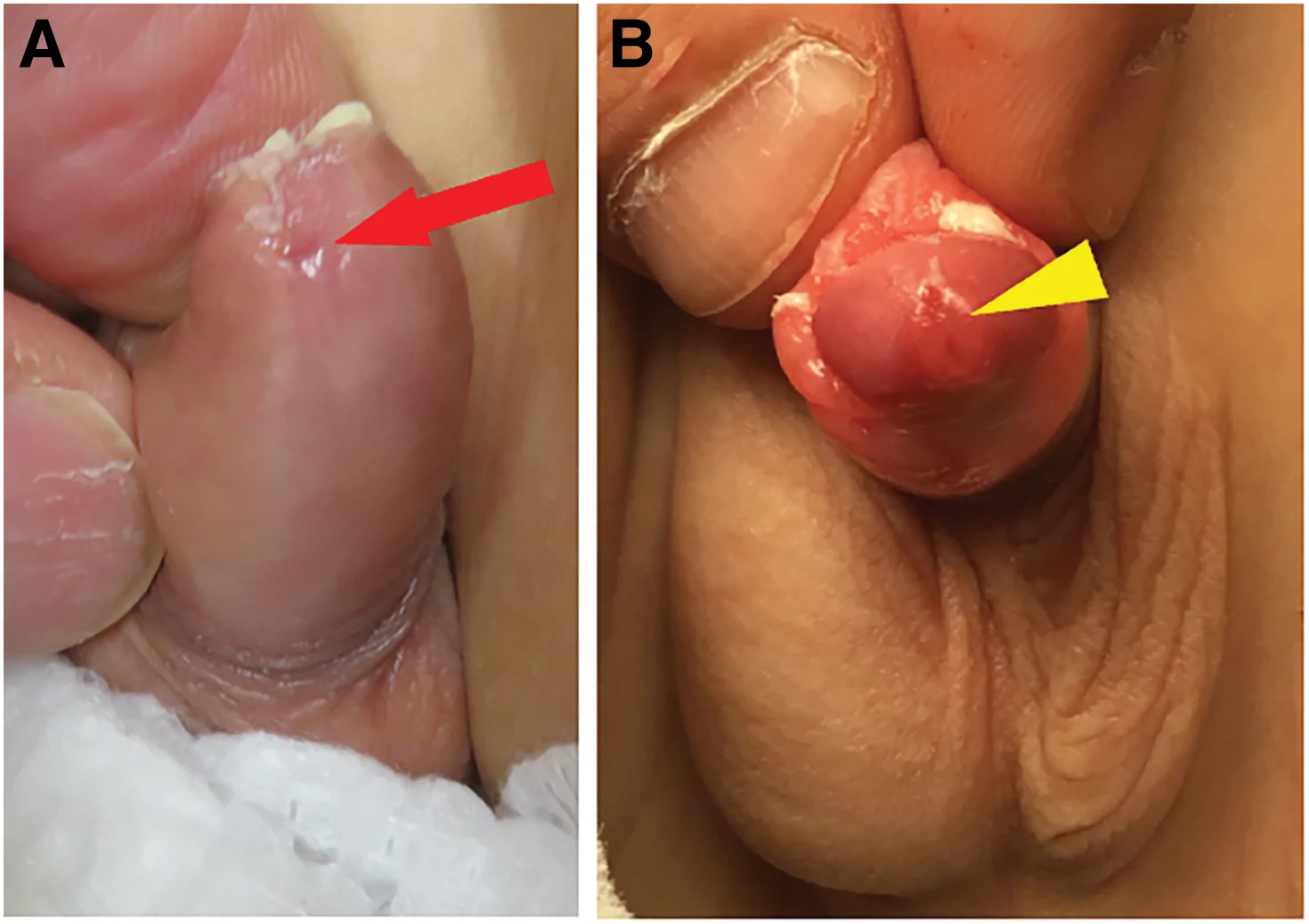
External genital findings. (**A**) Preputial adhesion, with the ectopic meatus in a hypospadiac position on the ventral penis (red arrow). (**B**) After glans exposure, the orthotopic meatus at the tip of the glans (yellow arrowhead).

To achieve cystostomy-free spontaneous voiding, a minimally invasive staged approach was employed between 12 and 15 months of age. This involved 2 sessions of endoscopic, guidewire-assisted balloon dilation performed via both the ectopic hypospadiac meatus and the cystostomy tract, combined with holmium:YAG laser ablation to the acquired PUD, a technique previously reported for acquired PUD after anorectoplasty.^8)^ At the 1st session, after the preputial adhesion had been released, only a single meatus was apparent at the tip of the glans. Intraoperative urethrography through the cystostomy showed that voiding occurred not through this glanular meatus but through a ventral midline opening just proximal to the coronal sulcus; the 2 external meati were thereby identified for the first time (**Fig. 3A** and **3B**). Urethroscopy through the glanular meatus revealed a stenosis so tight that a guidewire could not be passed, confirming it to be the dorsal channel. The ventral channel was therefore identified as the functional main urethra, and its stenotic segment was dilated through the hypospadiac meatus, after which a 6-Fr Foley catheter was left in place. Laser ablation was applied to the mucosa of the acquired PUD. At the 2nd session, ablation was considered complete once the PUD was no longer visualized on intraoperative urethrography. The dilation improved the patency of the functional ventral channel and enabled spontaneous voiding.

A graded clamping regimen (6- to 12-hour daily clamping) was then introduced, and spontaneous voiding through the ventral urethra was confirmed. However, at 19 months of age, a few days before a planned admission for VCUG-guided assessment for cystostomy removal, the patient developed a single febrile urinary tract infection, treated with oral cefdinir and subsequent trimethoprim–sulfamethoxazole prophylaxis; contrast imaging was deferred until the infection had resolved. The cystostomy was ultimately removed at 21 months of age, when VCUG showed voiding through the ventral channel with complete bladder emptying and no post-void residual urine, with an estimated bladder capacity of at least 30 mL (**Fig. 1C**). A residual PUD was again visualized and measured 11.7 × 6.1 mm.

**Fig. 4:**
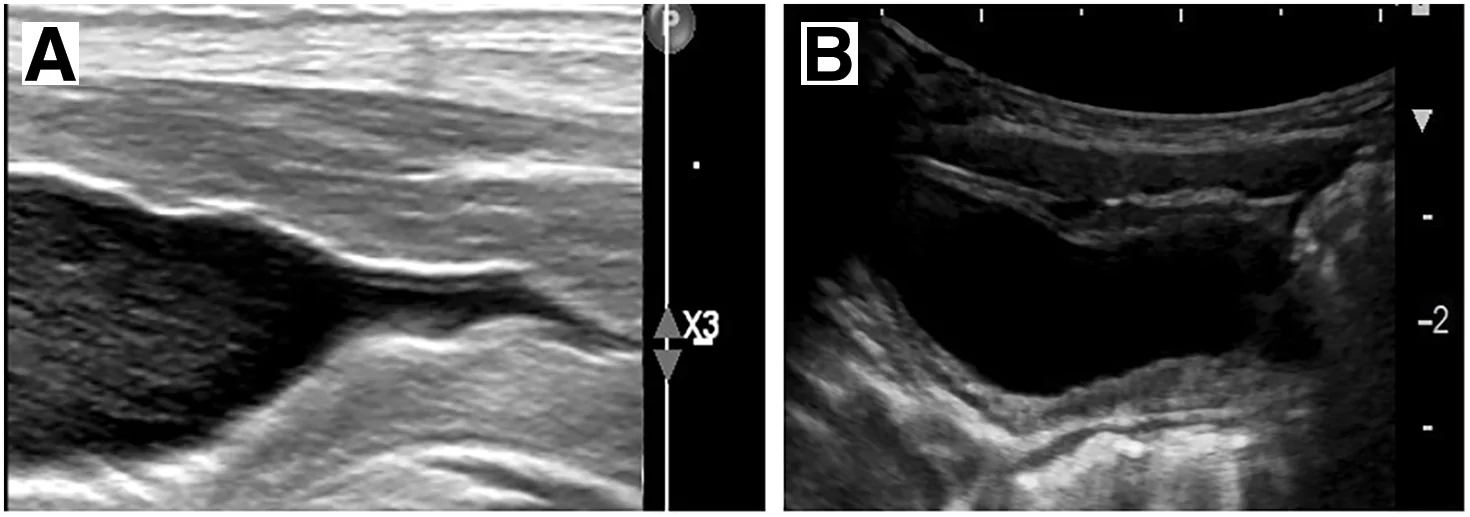
Bladder ultrasonography imaging. (**A**) Postnatal day 1: no bladder-wall thickening. (**B**) Most recent evaluation (28 months of age): bladder-wall thickness approximately 6 mm without trabeculation.

At the most recent follow-up (age 28 months, 7 months after cystostomy removal), the patient maintains spontaneous voiding with preserved renal function. The serum creatinine was 0.23 mg/dL and the blood urea nitrogen was 9.9 mg/dL. Bladder ultrasonography showed a bladder-wall thickness of about 6 mm without trabeculation and no hydronephrosis (**Fig. 4A** and **4B**). The small residual PUD has remained asymptomatic, and a further session of holmium:YAG laser ablation is planned. Repair of hypospadias, neurosurgical repair of the terminal filar lipoma, and bilateral orchiopexy for the undescended testes are also planned.

## DISCUSSION

The Effmann classification divides urethral duplication into incomplete (type I) and complete (type II) forms. Among complete duplications, the 2 urethras may arise separately from the bladder (type IIA1) or from a common origin (type IIA2), and in the Y-subtype (type IIA2-Y) the accessory ventral channel opens onto the perineum, anal canal, or rectum.^2)^ ARM is not evenly distributed across these types but is concentrated in the complete forms: in a systematic review of 250 boys, an associated ARM was recorded in 6 of 55 type IIA2 and 7 of 91 type IIA2-Y cases, but in only 1 of 38 type I and 1 of 21 type IIA1 cases.^1)^ The predilection of the Y-subtype for ARM has an embryological explanation: defective partitioning of the hindgut and the urogenital sinus by the urorectal septum produces both the anorectal anomaly and an accessory ventral urethra that communicates with the anorectum.^9)^ Consistent with this, an associated ARM was present in 7 of 10 boys in a series of complete Y-type duplications.^10)^ Our patient had a type IIA2 duplication without a Y-configuration: the ventral channel opened on the ventral surface of the penis rather than into the anorectum, and the rectourethral fistula was a separate feature of the intermediate ARM.

In both isolated and ARM-associated duplications, the ventral channel is characteristically the functional one. Effmann et al., in their original series, found the accessory ventral channel to be the better-functioning urethra in every patient, regardless of the position of its external meatus,^2)^ and in a series of Y-type duplications, 17 of 18 boys voided mainly through the ventral channel.^3)^ The dorsal orthotopic urethra, by contrast, is usually narrow or hypoplastic, and the degree of its patency forms the basis of the reconstructive classification proposed by Kurian et al.^3)^ Most boys therefore present with a double urinary stream, incontinence, recurrent urinary infection, or an abnormal 2nd meatus rather than with obstruction.^1)^ Stenosis of the ventral, functional channel is less common—reported in 8 of 91 type IIA2-Y cases—and, when both channels are stenotic or abortive, the bladder cannot empty and renal function may be compromised.^1)^ In our patient, the ventral channel was also stenotic, so that neither channel provided adequate drainage and complete urinary retention developed during the neonatal period; we identified and preserved the ventral channel as the functional urethra in accordance with this general principle while managing the coexisting stenosis of both channels.

Two aspects of this case deserve explanation: the lack of any antenatal abnormality and the abrupt onset of retention on postnatal day 4. *In utero*, the obstruction must have been incomplete. The ventral channel, although critically narrow, remained marginally patent, as shown by the spontaneous voiding about 2 hours after birth, and the fetal bladder therefore emptied well enough to avoid distension, upper tract dilatation, and oligohydramnios. The coexisting duodenal atresia, which caused a mild tendency to polyhydramnios, may also have masked any relative reduction in the fetal urinary contribution to the amniotic fluid. The onset on day 4 first suggested an iatrogenic cause because the retention was noticed the day after fentanyl was started, and opioids are well recognized to cause urinary retention.^11,12)^ However, the retention persisted after fentanyl was stopped, and the urethra still could not be catheterized. The opioid therefore unmasked the obstruction rather than causing it; presumably, an outlet that had only just been adequate failed once detrusor contraction was blunted.

Because neither channel could be catheterized, early percutaneous cystostomy served 2 purposes: secure decompression of the obstructed urinary tract and stable access for contrast studies, allowing accurate delineation of the lower urinary tract anatomy. A catheter-free surgical vesicostomy, which is often preferred in order to limit catheter-related infection, was judged less suitable here because endoscopic recanalization of the functional channel was considered achievable and would spare the child a second diversion and its later closure. This preoperative clarification of the functional ventral channel—and of its relationship to the rectourethral fistula and the prostatic utricle—guided operative planning.

During definitive anorectal reconstruction, dissecting the rectourethral fistula carries a risk of injuring the adjacent functional urethra. In our patient, the urethra was stenotic and a urethroscope could not be passed, so the fistula could not be confirmed from the urethral side. We therefore took particular care to divide and ligate the fistula on the rectal side, away from the urethra (**Fig. 2**). The postoperative VCUG showed that the ventral channel remained unchanged from its preoperative appearance and had been preserved. The trade-off of this urethra-sparing strategy is a retained fistula remnant, which presented as an acquired PUD—one of the commonest complications after ARM repair, and one that may harbor infection, calculi, and, rarely, malignancy.^13)^ There is a report in which an acquired diverticulum after posterior sagittal anorectoplasty in a boy with a Y-type duplication was managed endoscopically.^14)^ We regard such a diverticulum as an accepted and treatable consequence of protecting a duplicated urethra.

Following excision of the prostatic utricle, our patient demonstrated significant post-void residual urine, consistent with persistence of the pre-existing stenosis involving the functional ventral channel and the bulbar urethra. Guidewire-assisted balloon dilation through the hypospadiac ventral meatus and the cystostomy tract restored patency of the functional channel, and holmium:YAG laser ablation was applied to the acquired PUD. This laser ablation technique has been reported for acquired PUD after anorectoplasty.^8)^ The PUD was, however, demonstrated again at cystostomy removal, 6 months after the 2nd session, with a larger short axis than before ablation. Two explanations seem possible: either the intraoperative urethrography at the end of that session was falsely negative, or the resumption of urethral voiding after relief of the stenosis caused true interval enlargement.

Despite the residual PUD, the restored urethral patency allowed the cystostomy to be removed at 21 months of age without any open urethral reconstruction. In selected infants with complex anatomy, especially when other reconstructive procedures are already planned, such minimally invasive interventions may restore voiding while limiting morbidity.

Long-term follow-up remains essential. This patient has a terminal filar lipoma, for which neurosurgical repair is planned at another institution. Its effect on bladder function remains uncertain, and a neurogenic contribution to voiding cannot be entirely excluded. Nevertheless, the dominant obstruction was clearly anatomical and was relieved endoscopically, so a urodynamic study has been deferred and is planned together with the scheduled neurosurgical repair. Accordingly, voiding dynamics, upper urinary tract status, and the small residual diverticulum will require ongoing monitoring. This report has several limitations. It describes a single patient with relatively short follow-up. Post-void residual urine was not quantified before or between the endoscopic sessions, although VCUG at cystostomy removal (21 months of age) confirmed no post-void residual urine; bladder capacity was documented only at the time of cystostomy removal, and no urodynamic study or renal scintigraphy has yet been performed, so a neurogenic component and early renal scarring cannot be excluded. In summary, this case supports an anatomy-driven strategy for ARM associated with urethral duplication: complete preoperative delineation of the duplicated urethra and its relationship to the rectourethral fistula, preservation of the functional ventral channel by keeping the dissection on the rectal side, and stepwise endourologic management of the residual stenosis and of the acquired diverticulum that this strategy leaves behind.

## CONCLUSIONS

We report a neonate with Effmann type IIA2 urethral duplication coexisting with intermediate-type ARM, presenting with neonatal urinary retention due to stenosis of both urethral channels. Early percutaneous decompression allowed anatomic mapping, and the functional ventral channel was preserved during LAARP by keeping the fistula dissection on the rectal side. Stepwise, minimally invasive endourologic management of the residual stenosis and the acquired PUD achieved stable spontaneous voiding without extensive open urethral reconstruction. This case shows the importance of preserving the functional channel and of anatomy-based planning in ARM complicated by urethral duplication.

## DECLARATIONS

### Use of artificial intelligence (AI) tool

During the preparation of this work, the authors used ChatGPT (OpenAI) to improve language and readability and to assist with manuscript organization. After using this tool, the authors reviewed and edited the content as needed and take full responsibility for the content of the manuscript.

### Availability of data and materials

The data supporting the findings of this study are not publicly available due to patient privacy and confidentiality. De-identified data may be made available from the corresponding author upon reasonable request, subject to institutional requirements.
